# Crystal structure of (*E*)-2-[3-(*tert*-but­yl)-2-hy­droxy­benzyl­idene]-*N*-cyclo­hexyl­hydrazine-1-carbo­thio­amide

**DOI:** 10.1107/S2056989018013129

**Published:** 2018-09-21

**Authors:** Md. Azharul Arafath, Huey Chong Kwong, Farook Adam, Mohd. R. Razali

**Affiliations:** a Department of Chemistry, Shahjalal University of Science and Technology, Sylhet 3114, Bangladesh; b School of Chemical Sciences, Universiti Sains Malaysia, Penang 11800 USM, Malaysia

**Keywords:** crystal structure, cyclo­hexyl­hydrazine, soft Lewis base, carbo­thio­amide Schiff base

## Abstract

In the title compound, C_18_H_27_N_3_OS, the cyclo­hexane ring has a chair conformation. The azomethine C=N double bond has an *E* configuration. The nearly planar hydrazinecarbo­thio­amide moiety and substituted benzene ring are twisted by 31.13 (5)° relative to each other. The amide moiety and the cyclo­hexane ring are almost perpendicular to each other; a similar conformation was previously observed in reported structures. In the crystal, mol­ecules are linked by N—H⋯S hydrogen bonds, forming inversion dimers with an *R* ^2^_2_(8) ring motif.

## Abstract 2

The title compound is composed of cyclo­hexane and benzene rings connected through a hydrazinecarbo­thio­amide moiety. In the crystal, the mol­ecules are connected by N—H⋯S hydrogen bonds.

## Chemical context   

The thio­semicarbazone Schiff base is comprised of two soft Lewis bases – the sulfur and nitro­gen coordinating sites as well as a hard Lewis base – the oxygen atom (Mohamed *et al.*, 2009 ▸). Such Schiff bases are of special inter­est because of their specific coordinating ability to some metal ions (Arion *et al.*, 2001 ▸; Leovac & Češljević, 2002 ▸; Chandra & Sangeetika, 2004 ▸; Singh *et al.*, 2000 ▸; Gerbeleu *et al.*, 2008 ▸; Mohamed *et al.*, 2009 ▸). Several reports have highlighted the importance of the chelate metal complexes of thio­semicarbazone Schiff bases for medicinal applications, particularly against cancer (Paterson & Donnelly, 2011 ▸; Ziessel, 2001 ▸; Salam *et al.*, 2012 ▸; Arafath *et al.*, 2017*a* ▸). Thus thio­semicarbazones with ONS coordinating sites are important in coordination chemistry because of their strong bonding ability to transition metal ions as well as because of their pharmaceutical uses (Rayati *et al.*, 2007 ▸; Alomar *et al.*, 2009 ▸; Vieites *et al.*, 2009 ▸).
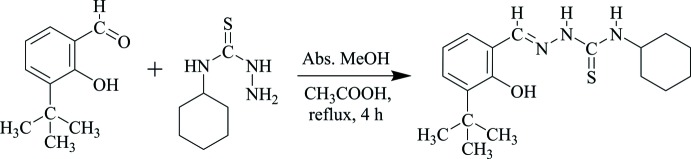


## Structural commentary   

**Figure 1:**
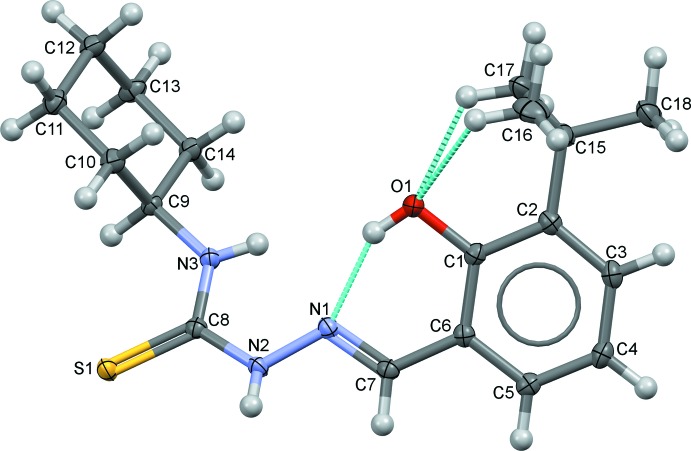
The mol­ecular structure with the atom-labelling scheme and displacement ellipsoids at the 50% probability level.

The title compound exhibits an *E* configuration with respect to the azomethine C=N double bond. The overall conformation of the title compound can be described by five torsion angles, τ1 [C1—C6—C7=N1; 11.80 (16)°] between the benzyl­idine ring and the azomethine double bond, τ2 [C7=N1—N2—C8; −170.08 (10)°] between the azomethine double bond and the hydrazine moiety, τ3 [N1—N2—C8—N3; 12.50 (15)°] between the hydrazine moiety and the carbo­thio group, τ4 [N2—C8—N3—C9; −176.16 (10)°] between the carbo­thio and amide groups and τ5 [C8—N3—C9—C10; 78.28 (13)°] between the amide group and the cyclo­hexane ring. In the previously reported related structure (*E*)-2-(5-chloro-2-hy­droxy­benzyl­idene)-*N*-cyclo­hexyl­hydrazine-1-carbo­thio­amide (OBOLOJ; Arafath, *et al.* 2017*b* ▸), values of τ1, τ2, τ3 and τ4 are −4.6 (3), −176.04 (17), −5.5 (3) and 176.67 (17)°, respectively]. The amide group and the cyclo­hexane ring are almost perpendic­ular to each other, with a τ5 torsion angle of −83.7 (2)°, possibly as a result of repulsion between the adjacent sulfur atom and the cyclo­hexane ring. In the mol­ecule, the hy­droxy group acts as both a hydrogen-bond acceptor and hydrogen-bond donor for the adjacent methyl and hydrazine groups, forming three intra­molecular hydrogen bonds with an *S*(6) ring motif (Table 1 ▸, Fig. 1 ▸).

## Supra­molecular features   

**Figure 2:**
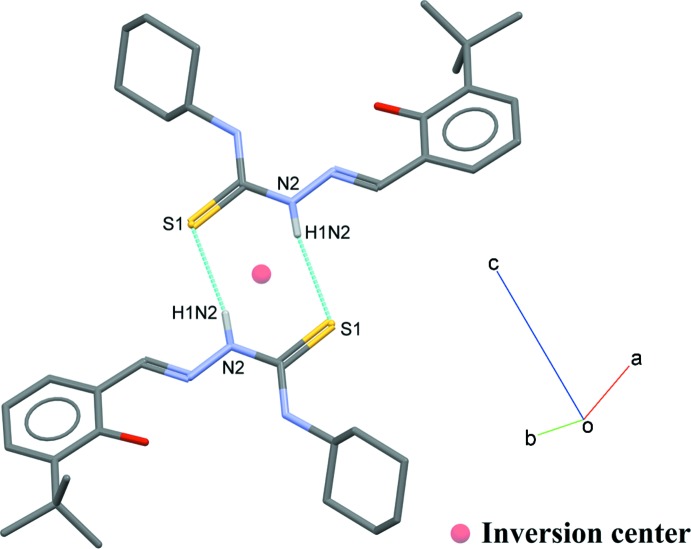
A view of a centrosymmetric dimer of C_18_H_27_N_3_OS with N2—-H1*N*2⋯S1 hydrogen bonds shown as cyan dotted lines. Hydrogen atoms not involved in with these inter­actions are omitted for clarity.

In the crystal, the mol­ecules are linked into inversion dimers *via* N—H⋯S hydrogen bond, forming an 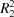
(8) ring motif (Fig. 2 ▸, Table 1 ▸).

## Database survey   

A search of the Cambridge Structural Database (CSD Version 5.39, last update February 2018; Groom *et al.*, 2016 ▸) using (*E*)-2-benzyl­idene-*N*-cyclo­hexyl­hydrazine-1-carbo­thio­amide as a reference moiety resulted in six structures containing the cyclo­hexlhydrazinecarbo­thio­amide moiety with different substituents. They include (*E*)-2-*X*-*N*-cyclo­hexyl­hydrazine-1-carbo­thio­amide, where *X* = 4-amino­benzyl­idene (BEVNAR; Koo *et al.*, 1981 ▸), 5-bromo-2-hy­droxy-3-meth­oxy­benzyl­idene (LAQCIR; Jacob & Kurup, 2012 ▸), anthracen-9-yl­methyl­ene (NALCOD; Basheer, Willis *et al.*, 2016 ▸), 5-chloro-2-hy­droxy­benzyl­idene (OBOLOJ; Arafath, *et al.* 2017*b* ▸), 4-eth­oxy­benzyl­idene (XOYKAZ; Bhat *et al.*, 2015 ▸) and (2-hydroxynaphthalen-1-yl)methyl­ene (BEFZIY; Basheer, Bhuvanesh *et al.*, 2016 ▸). In these six compounds, the torsion angles between benzyl­idene ring and the hydrazinecarbo­thio­amide moiety range from 4.70 to 36.40°. In comparison, torsion angle τ5 has values close to 90° for all compounds Table 2 ▸).

## Synthesis and crystallization   

3-(*tert*-But­yl)-2-hy­droxy­benzaldehyde (0.89 g, 5.00 mmol) was dissolved in 20.0 mL of methanol. Glacial acetic acid (0.20 mL) was added, and the mixture was refluxed for 30 minutes. *N*-Cyclo­hexyl­hydrazinecarbo­thio­amide (0.87 g, 5.00 mmol) in 20.0 mL methanol was then added dropwise with stirring to the aldehyde solution. The resulting colourless solution was refluxed for 4 h with stirring. The colourless precipitate that formed was filtered off and washed with 5.0 mL ethanol and 5.0 mL *n*-hexane. The recovered product was dissolved in acetone for recrystallization. Colourless single crystals suitable for X-ray diffraction was obtained on slow evaporation of the solvent (m.p. 502–503 K, yield 98%). Analysis calculated for C_18_H_27_N_3_OS (FW: 333.49 g mol^−1^); C, 64.77; H, 8.10; N, 12.60; found: C, 64.73; H, 8.10; N, 12.65%. ^1^H NMR (500 MHz, DMSO-*d*_6_, Me_4_Si ppm): δ 11.23 (*s*, 1.0, N—NH), δ 10.23 (*s*, 1.0, OH), δ 8.27 (*s*, 1.0, HC=N), δ 8.09 (*d*, *J* = 8.00 Hz, 1.0, SC=NH), δ 7.26–6.87 (multiplet, 2.96, aromatic-H), δ 1.39 [*s*, 9.0, Ph—C(CH_3_)_3_], δ 1.88–1.15 (multiplet, 11.0, cyclo­hexyl-H). ^13^C NMR (DMSO-*d*_6_, Me_4_Si ppm): δ 176.05 (C=S), δ 155.31 (C=N), δ 146.32–119.03 (C-aromatic), δ 29.40 (CH_3_), δ 53.04–24.85 (C-cyclo­hex­yl). IR (KBr pellets υ_max_/cm^−1^): 3383 υ(N—NH), 3106 υ(OH), 2929 and 2854 υ(CH, cyclo­hex­yl), 1598 υ(C=N), 1536 υ(C=C, aromatic), 1299 υ (C-H, *sp^3^*, bend), 1258 υ(C=S).

## Refinement   

Crystal data, data collection and structure refinement details are summarized in Table 3 ▸. C-bound H atoms were positioned geometrically [C—H = 0.95–0.99 Å] and refined using a riding model with *U*_iso_(H) = 1.2 or 1.5*U*_eq_(C). All N- and O-bound H atoms were located from a difference-Fourier map and freely refined.

## Supplementary Material

Crystal structure: contains datablock(s) I. DOI: 10.1107/S2056989018013129/ld2145sup1.cif

Structure factors: contains datablock(s) I. DOI: 10.1107/S2056989018013129/ld2145Isup2.hkl

Supporting information file. DOI: 10.1107/S2056989018013129/ld2145Isup3.cml

CCDC reference: 1435681

Additional supporting information:  crystallographic information; 3D view; checkCIF report

## Figures and Tables

**Table 1:** Hydrogen-bond geometry (Å, °)

*D*—H⋯*A*	*D*—H	H⋯*A*	*D*⋯*A*	*D*—H⋯*A*
O1—H1*O*1⋯N1	0.834 (19)	1.901 (19)	2.6545 (12)	149.6 (19)
C16—H16*A*⋯O1	0.98	2.46	3.0774 (16)	121
C17—H17*C*⋯O1	0.98	2.26	2.9101 (15)	123
N2—H1*N*2⋯S1^i^	0.833 (14)	2.459 (14)	3.2779 (11)	165.6 (13)

Symmetry code: (i) 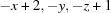.

**Table 2:** Selected dihedral and torsion angles (°) Dihedral is the dihedral angle between the mean planes of the benzyl­idene ring and the hydrazinecarbo­thio­amide moiety. τ5 is the C8—N3—C9—C10 torsion angle.

Compound	Dihedral	τ5
Title compound	31.13 (5)	78.32
BEVNAR	28.50	94.47
LAQCIR	16.64	86.22
NALKOD	22.00, 36.40	79.01, 79.19
OBOLOJ	6.92	83.70
XOYKAZ	12.72	85.82
BEFZIY	4.70	83.42

**Table 3:** Experimental details

Crystal data	
Chemical formula	C_18_H_27_N_3_OS
*M*_r_	333.48
Crystal system, space group	Monoclinic, *P*2_1_/*c*
Temperature (K)	100
*a*, *b*, *c* (Å)	13.4168 (6), 6.6070 (3), 20.5831 (9)
β (°)	93.032 (1)
*V* (Å^3^)	1822.03 (14)
*Z*	4
Radiation type	Mo *K*α
μ (mm^−1^)	0.19
Crystal size (mm)	0.57 × 0.30 × 0.29

Data collection	
Diffractometer	Bruker APEXII DUO CCD area-detector diffractometer
Absorption correction	Multi-scan (*SADABS*; Bruker, 2012 ▸
*T*_min_, *T*_max_	0.774, 0.879
No. of measured, independent and observed [*I* > 2σ(*I*)] reflections	39751, 4195, 3819
*R*_int_	0.031
(sin θ/λ)_max_ (Å^−1^)	0.650

Refinement	
*R*[*F* ^2^ > 2σ(*F* ^2^)], *wR*(*F* ^2^), *S*	0.032, 0.086, 1.04
No. of reflections	4195
No. of parameters	223
H-atom treatment	H atoms treated by a mixture of independent and constrained refinement
Δρ_max_, Δρ_min_ (e Å^−3^)	0.38, −0.22

Computer programs: *APEX2* and *SAINT* (Bruker, 2012 ▸), *SHELXS97* (Sheldrick, 2008 ▸), *Mercury* (Macrae *et al.*, 2006 ▸), *SHELX2013* (Sheldrick, 2015 ▸) and *PLATON* (Spek, 2009 ▸).

## supplementary crystallographic information

### Crystal data

**Table:**

C_18_H_27_N_3_OS	*F*(000) = 720
*M**_r_* = 333.48	*D*_x_ = 1.216 Mg m^−^^3^
Monoclinic, *P*2_1_/*c*	Mo *K*α radiation, λ = 0.71073 Å
*a* = 13.4168 (6) Å	Cell parameters from 9744 reflections
*b* = 6.6070 (3) Å	θ = 2.4–32.8°
*c* = 20.5831 (9) Å	µ = 0.19 mm^−^^1^
β = 93.032 (1)°	*T* = 100 K
*V* = 1822.03 (14) Å^3^	Block, colourless
*Z* = 4	0.57 × 0.30 × 0.29 mm

### Data collection

**Table:**

Bruker APEXII DUO CCD area-detector diffractometer	4195 independent reflections
Radiation source: fine-focus sealed tube	3819 reflections with *I* > 2σ(*I*)
Graphite monochromator	*R*_int_ = 0.031
φ and ω scans	θ_max_ = 27.5°, θ_min_ = 2.0°
Absorption correction: multi-scan (SADABS; Bruker, 2012	*h* = −17→17
*T*_min_ = 0.774, *T*_max_ = 0.879	*k* = −8→8
39751 measured reflections	*l* = −25→26

### Refinement

**Table:**

Refinement on *F*^2^	0 restraints
Least-squares matrix: full	Hydrogen site location: mixed
*R*[*F*^2^ > 2σ(*F*^2^)] = 0.032	H atoms treated by a mixture of independent and constrained refinement
*wR*(*F*^2^) = 0.086	*w* = 1/[σ^2^(*F*_o_^2^) + (0.0422*P*)^2^ + 0.8449*P*] where *P* = (*F*_o_^2^ + 2*F*_c_^2^)/3
*S* = 1.04	(Δ/σ)_max_ = 0.002
4195 reflections	Δρ_max_ = 0.38 e Å^−^^3^
223 parameters	Δρ_min_ = −0.22 e Å^−^^3^

### Special details

**Table:**

Experimental. The following wavelength and cell were deduced by SADABS from the direction cosines etc. They are given here for emergency use only: CELL 0.71104 13.523 6.653 20.749 89.939 93.047 89.965
Geometry. All esds (except the esd in the dihedral angle between two l.s. planes) are estimated using the full covariance matrix. The cell esds are taken into account individually in the estimation of esds in distances, angles and torsion angles; correlations between esds in cell parameters are only used when they are defined by crystal symmetry. An approximate (isotropic) treatment of cell esds is used for estimating esds involving l.s. planes.

### Fractional atomic coordinates and isotropic or equivalent isotropic displacement parameters (Å^2^)

**Table:**

	*x*	*y*	*z*	*U*_iso_*/*U*_eq_	
S1	1.02419 (2)	−0.15365 (4)	0.40715 (2)	0.01701 (8)	
O1	0.65801 (6)	0.39888 (12)	0.39017 (4)	0.02153 (18)	
N1	0.84612 (7)	0.31101 (14)	0.42569 (4)	0.01542 (18)	
N2	0.92566 (7)	0.17823 (14)	0.43545 (5)	0.01565 (19)	
N3	0.88665 (7)	0.04928 (14)	0.33416 (4)	0.01665 (19)	
C1	0.67407 (8)	0.57899 (16)	0.42115 (5)	0.0155 (2)	
C2	0.59978 (8)	0.72977 (17)	0.41452 (5)	0.0164 (2)	
C3	0.61893 (8)	0.91139 (17)	0.44745 (5)	0.0189 (2)	
H3A	0.5707	1.0165	0.4433	0.023*	
C4	0.70586 (8)	0.94540 (17)	0.48615 (5)	0.0190 (2)	
H4A	0.7155	1.0707	0.5082	0.023*	
C5	0.77763 (8)	0.79630 (16)	0.49220 (5)	0.0164 (2)	
H5A	0.8366	0.8182	0.5189	0.020*	
C6	0.76359 (8)	0.61241 (16)	0.45906 (5)	0.0145 (2)	
C7	0.84439 (8)	0.46494 (16)	0.46392 (5)	0.0151 (2)	
H7A	0.8971	0.4829	0.4961	0.018*	
C8	0.93944 (8)	0.03134 (16)	0.39073 (5)	0.0148 (2)	
C9	0.88503 (8)	−0.09682 (16)	0.28052 (5)	0.0145 (2)	
H9A	0.9545	−0.1468	0.2754	0.017*	
C10	0.81847 (8)	−0.27750 (17)	0.29436 (5)	0.0185 (2)	
H10A	0.7505	−0.2291	0.3028	0.022*	
H10B	0.8454	−0.3488	0.3338	0.022*	
C11	0.81297 (9)	−0.42424 (17)	0.23685 (6)	0.0213 (2)	
H11A	0.8797	−0.4841	0.2316	0.026*	
H11B	0.7663	−0.5356	0.2459	0.026*	
C12	0.77785 (9)	−0.31813 (17)	0.17386 (6)	0.0206 (2)	
H12A	0.7080	−0.2728	0.1771	0.025*	
H12B	0.7798	−0.4147	0.1372	0.025*	
C13	0.84377 (9)	−0.13575 (17)	0.16058 (5)	0.0200 (2)	
H13A	0.8166	−0.0644	0.1212	0.024*	
H13B	0.9119	−0.1831	0.1521	0.024*	
C14	0.84919 (8)	0.01120 (16)	0.21804 (5)	0.0175 (2)	
H14A	0.8956	0.1230	0.2090	0.021*	
H14B	0.7824	0.0703	0.2236	0.021*	
C15	0.50170 (8)	0.69323 (18)	0.37398 (6)	0.0212 (2)	
C16	0.44095 (10)	0.5302 (2)	0.40747 (7)	0.0339 (3)	
H16A	0.4784	0.4028	0.4090	0.051*	
H16B	0.3771	0.5102	0.3829	0.051*	
H16C	0.4285	0.5737	0.4518	0.051*	
C17	0.52133 (10)	0.6286 (2)	0.30399 (6)	0.0300 (3)	
H17A	0.5615	0.7323	0.2835	0.045*	
H17B	0.4576	0.6128	0.2790	0.045*	
H17C	0.5574	0.4997	0.3048	0.045*	
C18	0.43846 (10)	0.8868 (2)	0.36863 (6)	0.0290 (3)	
H18A	0.4767	0.9941	0.3484	0.043*	
H18B	0.4211	0.9298	0.4122	0.043*	
H18C	0.3773	0.8600	0.3419	0.043*	
H1N2	0.9483 (11)	0.161 (2)	0.4737 (7)	0.020 (3)*	
H1O1	0.7098 (14)	0.330 (3)	0.3961 (9)	0.043 (5)*	
H1N3	0.8464 (11)	0.149 (2)	0.3316 (7)	0.025 (4)*	

### Atomic displacement parameters (Å^2^)

**Table:**

	*U*^11^	*U*^22^	*U*^33^	*U*^12^	*U*^13^	*U*^23^
S1	0.01723 (14)	0.01842 (14)	0.01519 (14)	0.00573 (10)	−0.00098 (10)	−0.00137 (9)
O1	0.0196 (4)	0.0160 (4)	0.0282 (4)	0.0037 (3)	−0.0070 (3)	−0.0059 (3)
N1	0.0151 (4)	0.0153 (4)	0.0158 (4)	0.0025 (3)	0.0001 (3)	0.0010 (3)
N2	0.0162 (4)	0.0168 (4)	0.0135 (4)	0.0049 (3)	−0.0025 (3)	−0.0005 (3)
N3	0.0192 (4)	0.0151 (4)	0.0153 (4)	0.0045 (4)	−0.0023 (3)	−0.0021 (3)
C1	0.0177 (5)	0.0139 (5)	0.0152 (5)	−0.0003 (4)	0.0017 (4)	−0.0002 (4)
C2	0.0156 (5)	0.0185 (5)	0.0152 (5)	0.0019 (4)	0.0023 (4)	0.0021 (4)
C3	0.0197 (5)	0.0166 (5)	0.0207 (5)	0.0041 (4)	0.0048 (4)	0.0012 (4)
C4	0.0231 (5)	0.0148 (5)	0.0195 (5)	−0.0004 (4)	0.0045 (4)	−0.0025 (4)
C5	0.0172 (5)	0.0172 (5)	0.0151 (5)	−0.0022 (4)	0.0026 (4)	−0.0005 (4)
C6	0.0161 (5)	0.0144 (5)	0.0132 (5)	0.0009 (4)	0.0026 (4)	0.0014 (4)
C7	0.0150 (5)	0.0160 (5)	0.0142 (5)	−0.0001 (4)	0.0002 (4)	0.0012 (4)
C8	0.0140 (5)	0.0146 (5)	0.0158 (5)	−0.0007 (4)	0.0015 (4)	−0.0002 (4)
C9	0.0155 (5)	0.0144 (5)	0.0137 (5)	0.0009 (4)	0.0002 (4)	−0.0016 (4)
C10	0.0205 (5)	0.0180 (5)	0.0170 (5)	−0.0018 (4)	0.0006 (4)	0.0023 (4)
C11	0.0251 (6)	0.0153 (5)	0.0233 (6)	−0.0024 (4)	−0.0012 (4)	−0.0005 (4)
C12	0.0228 (5)	0.0203 (5)	0.0183 (5)	−0.0030 (4)	−0.0020 (4)	−0.0033 (4)
C13	0.0242 (6)	0.0218 (5)	0.0140 (5)	−0.0031 (4)	0.0006 (4)	−0.0012 (4)
C14	0.0215 (5)	0.0153 (5)	0.0156 (5)	−0.0016 (4)	−0.0005 (4)	0.0010 (4)
C15	0.0168 (5)	0.0252 (6)	0.0211 (6)	0.0049 (4)	−0.0022 (4)	−0.0007 (4)
C16	0.0194 (6)	0.0392 (7)	0.0422 (8)	−0.0066 (5)	−0.0058 (5)	0.0052 (6)
C17	0.0263 (6)	0.0391 (7)	0.0237 (6)	0.0126 (5)	−0.0071 (5)	−0.0079 (5)
C18	0.0237 (6)	0.0352 (7)	0.0274 (6)	0.0138 (5)	−0.0035 (5)	−0.0036 (5)

### Geometric parameters (Å, º)

**Table:**

S1—C8	1.6912 (11)	C10—H10A	0.9900
O1—C1	1.3619 (13)	C10—H10B	0.9900
O1—H1O1	0.836 (19)	C11—C12	1.5264 (16)
N1—C7	1.2867 (14)	C11—H11A	0.9900
N1—N2	1.3879 (12)	C11—H11B	0.9900
N2—C8	1.3571 (14)	C12—C13	1.5279 (16)
N2—H1N2	0.836 (15)	C12—H12A	0.9900
N3—C8	1.3357 (14)	C12—H12B	0.9900
N3—C9	1.4659 (13)	C13—C14	1.5290 (15)
N3—H1N3	0.854 (16)	C13—H13A	0.9900
C1—C2	1.4107 (15)	C13—H13B	0.9900
C1—C6	1.4145 (15)	C14—H14A	0.9900
C2—C3	1.3953 (16)	C14—H14B	0.9900
C2—C15	1.5394 (15)	C15—C18	1.5354 (16)
C3—C4	1.3953 (16)	C15—C16	1.5359 (18)
C3—H3A	0.9500	C15—C17	1.5384 (17)
C4—C5	1.3786 (15)	C16—H16A	0.9800
C4—H4A	0.9500	C16—H16B	0.9800
C5—C6	1.4012 (15)	C16—H16C	0.9800
C5—H5A	0.9500	C17—H17A	0.9800
C6—C7	1.4571 (14)	C17—H17B	0.9800
C7—H7A	0.9500	C17—H17C	0.9800
C9—C14	1.5260 (14)	C18—H18A	0.9800
C9—C10	1.5265 (15)	C18—H18B	0.9800
C9—H9A	1.0000	C18—H18C	0.9800
C10—C11	1.5288 (16)		
			
C1—O1—H1O1	107.5 (12)	C10—C11—H11A	109.3
C7—N1—N2	116.72 (9)	C12—C11—H11B	109.3
C8—N2—N1	119.02 (9)	C10—C11—H11B	109.3
C8—N2—H1N2	119.2 (10)	H11A—C11—H11B	108.0
N1—N2—H1N2	117.3 (10)	C11—C12—C13	111.02 (9)
C8—N3—C9	125.75 (9)	C11—C12—H12A	109.4
C8—N3—H1N3	115.2 (10)	C13—C12—H12A	109.4
C9—N3—H1N3	118.7 (10)	C11—C12—H12B	109.4
O1—C1—C2	118.57 (9)	C13—C12—H12B	109.4
O1—C1—C6	120.22 (9)	H12A—C12—H12B	108.0
C2—C1—C6	121.21 (10)	C12—C13—C14	111.51 (9)
C3—C2—C1	116.69 (10)	C12—C13—H13A	109.3
C3—C2—C15	121.90 (10)	C14—C13—H13A	109.3
C1—C2—C15	121.40 (10)	C12—C13—H13B	109.3
C2—C3—C4	122.87 (10)	C14—C13—H13B	109.3
C2—C3—H3A	118.6	H13A—C13—H13B	108.0
C4—C3—H3A	118.6	C9—C14—C13	110.78 (9)
C5—C4—C3	119.68 (10)	C9—C14—H14A	109.5
C5—C4—H4A	120.2	C13—C14—H14A	109.5
C3—C4—H4A	120.2	C9—C14—H14B	109.5
C4—C5—C6	120.02 (10)	C13—C14—H14B	109.5
C4—C5—H5A	120.0	H14A—C14—H14B	108.1
C6—C5—H5A	120.0	C18—C15—C16	108.16 (10)
C5—C6—C1	119.49 (10)	C18—C15—C17	106.58 (10)
C5—C6—C7	117.78 (9)	C16—C15—C17	110.30 (11)
C1—C6—C7	122.71 (10)	C18—C15—C2	111.19 (10)
N1—C7—C6	121.60 (9)	C16—C15—C2	108.99 (9)
N1—C7—H7A	119.2	C17—C15—C2	111.55 (9)
C6—C7—H7A	119.2	C15—C16—H16A	109.5
N3—C8—N2	116.29 (9)	C15—C16—H16B	109.5
N3—C8—S1	123.94 (8)	H16A—C16—H16B	109.5
N2—C8—S1	119.70 (8)	C15—C16—H16C	109.5
N3—C9—C14	108.55 (9)	H16A—C16—H16C	109.5
N3—C9—C10	111.09 (9)	H16B—C16—H16C	109.5
C14—C9—C10	111.20 (9)	C15—C17—H17A	109.5
N3—C9—H9A	108.6	C15—C17—H17B	109.5
C14—C9—H9A	108.6	H17A—C17—H17B	109.5
C10—C9—H9A	108.6	C15—C17—H17C	109.5
C9—C10—C11	110.87 (9)	H17A—C17—H17C	109.5
C9—C10—H10A	109.5	H17B—C17—H17C	109.5
C11—C10—H10A	109.5	C15—C18—H18A	109.5
C9—C10—H10B	109.5	C15—C18—H18B	109.5
C11—C10—H10B	109.5	H18A—C18—H18B	109.5
H10A—C10—H10B	108.1	C15—C18—H18C	109.5
C12—C11—C10	111.50 (9)	H18A—C18—H18C	109.5
C12—C11—H11A	109.3	H18B—C18—H18C	109.5
			
C7—N1—N2—C8	−170.08 (10)	C9—N3—C8—S1	6.94 (16)
O1—C1—C2—C3	179.54 (10)	N1—N2—C8—N3	12.50 (15)
C6—C1—C2—C3	−0.44 (15)	N1—N2—C8—S1	−170.46 (8)
O1—C1—C2—C15	0.70 (15)	C8—N3—C9—C14	−159.15 (10)
C6—C1—C2—C15	−179.27 (10)	C8—N3—C9—C10	78.28 (13)
C1—C2—C3—C4	−0.95 (16)	N3—C9—C10—C11	177.21 (9)
C15—C2—C3—C4	177.88 (10)	C14—C9—C10—C11	56.19 (12)
C2—C3—C4—C5	0.87 (17)	C9—C10—C11—C12	−55.62 (12)
C3—C4—C5—C6	0.64 (16)	C10—C11—C12—C13	55.03 (13)
C4—C5—C6—C1	−1.98 (16)	C11—C12—C13—C14	−55.10 (13)
C4—C5—C6—C7	176.29 (10)	N3—C9—C14—C13	−178.71 (9)
O1—C1—C6—C5	−178.09 (9)	C10—C9—C14—C13	−56.20 (12)
C2—C1—C6—C5	1.89 (16)	C12—C13—C14—C9	55.70 (12)
O1—C1—C6—C7	3.74 (16)	C3—C2—C15—C18	7.08 (15)
C2—C1—C6—C7	−176.29 (10)	C1—C2—C15—C18	−174.15 (10)
N2—N1—C7—C6	−178.82 (9)	C3—C2—C15—C16	−112.06 (12)
C5—C6—C7—N1	−166.41 (10)	C1—C2—C15—C16	66.72 (14)
C1—C6—C7—N1	11.80 (16)	C3—C2—C15—C17	125.90 (12)
C9—N3—C8—N2	−176.16 (10)	C1—C2—C15—C17	−55.32 (14)

### Hydrogen-bond geometry (Å, º)

**Table:**

*D*—H···*A*	*D*—H	H···*A*	*D*···*A*	*D*—H···*A*
O1—H1*O*1···N1	0.834 (19)	1.901 (19)	2.6545 (12)	149.6 (19)
C16—H16*A*···O1	0.98	2.46	3.0774 (16)	121
C17—H17*C*···O1	0.98	2.26	2.9101 (15)	123
N2—H1*N*2···S1^i^	0.833 (14)	2.459 (14)	3.2779 (11)	165.6 (13)

Symmetry code: (i) −*x*+2, −*y*, −*z*+1.
